# Mesenchymal stem cell extracellular vesicle vascularization bioactivity and production yield are responsive to cell culture substrate stiffness

**DOI:** 10.1002/btm2.10743

**Published:** 2025-01-07

**Authors:** Emily H. Powsner, Stephanie M. Kronstadt, Kristin Nikolov, Amaya Aranda, Steven M. Jay

**Affiliations:** ^1^ Fischell Department of Bioengineering University of Maryland College Park Maryland USA; ^2^ Program in Molecular Biology University of Maryland College Park Maryland USA

**Keywords:** cell‐derived therapy, exosome, mesenchymal stromal cell

## Abstract

Mesenchymal stem cell‐derived extracellular vesicles (MSC EVs) are an attractive therapeutic option for regenerative medicine applications due to their inherently pro‐angiogenic and anti‐inflammatory properties. However, reproducible and cost‐effective production of highly potent therapeutic MSC EVs is challenging, limiting their translational potential. Here, we investigated whether the well‐characterized responsiveness of MSCs to their mechanical environment—specifically, substrate stiffness—could be exploited to generate EVs with increased therapeutic bioactivity without the need for biochemical priming or genetic manipulation. Using polydimethylsiloxane and bone marrow‐derived MSCs (BM‐MSCs), we show that decreasing the stiffness of MSC substrates to as low as 3 kPa significantly improves the pro‐angiogenic bioactivity of EVs as measured by tube formation and gap closure assays. We also demonstrate that lower substrate stiffness improves EV production and overall yield, important for clinical translation. Furthermore, we establish the mechanoresponsiveness of induced pluripotent stem cell‐derived MSC (iMSC) EVs and their comparability to BM‐MSC EVs, again using tube formation and gap closure assays. With this data, we confirm iMSCs' feasibility as an alternative, renewable cell source for EV production with reduced donor variability. Overall, these results suggest that utilizing substrate stiffness is a promising, simple, and a potentially scalable approach that does not require exogenous cargo or extraneous reagents to generate highly potent pro‐angiogenic MSC EVs.

**Translational Impact Statement**

Mesenchymal stem cell‐derived extracellular vesicles (MSC EVs) are an attractive therapeutic option for regenerative medicine applications. However, there are major biomanufacturing and clinical translation challenges for MSC EVs, including: (1) lower‐than‐desired potency that necessitates higher and/or more doses to achieve therapeutic outcomes, thus increasing safety risks; and (2) donor variability and the finite expansion capabilities of primary MSCs, which limits reproducibility and large‐scale production potential. This manuscript reports two important advances that promote translational utility of MSC EVs. We demonstrate that substrate stiffness can be exploited to generate MSC EVs with increased therapeutic bioactivity without the need for biochemical priming or genetic manipulation. We further show that this outcome can be achieved using induced pluripotent stem cell‐derived MSC EVs, setting the stage for reproducible production of high potency regenerative medicine products.

## 1 INTRODUCTION

Mesenchymal stem cell‐derived extracellular vesicles (MSC EVs) have gained significant interest within the field of regenerative medicine based on their pro‐angiogenic, anti‐inflammatory, and anti‐apoptotic properties. MSCs are the most common source of EVs investigated in therapeutic clinical trials^1^ and MSC EV hold promise for therapeutic applications such as chronic wounds, cardiovascular diseases, and inflammatory diseases^2^ and have been safely and successfully used in humans.^3^ However, despite their widespread potential and growing traction pre‐clinically and in clinical trials, successful FDA approval and ultimate clinical translation have not yet been achieved. This can be attributed to numerous challenges that MSC EVs and EVs in general face that include, but are not limited to, low potency and donor variability.^4^, ^5^

Most common approaches to overcoming low potency issues include exogenous and endogenous loading of therapeutic cargo.^6^ Numerous groups have demonstrated that overexpressing therapeutic microRNAs (miRNAs) via genetic engineering (endogenous loading) or exogenously loading post‐EV isolation (e.g., sonication and electroporation) is able to improve their efficacy. For example, by overexpressing miR‐181a, a miRNA implicated in immune regulation, Wei et al.^7^ showed that the MSC EVs were able to exert a significantly stronger effect in reducing inflammation and improving cardiac function in mice after ischemia–reperfusion injury compared to native MSC EVs. Another common approach is to precondition the cells with cytokines, growth factors, and/or hypoxia to promote certain therapeutic effects.^8^, ^9^ While these methods are effective, they require expensive extraneous reagents that, at best, necessitate more intensive, costly downstream purification, and, at worst, may be left behind at levels high enough to affect EV bioactivity and experimental outcomes, posing additional challenges to clinical translation.^10^, ^11^ Alternatively, researchers have shown that mechanical stimuli such as substrate stiffness, stretch and compression, and fluid shear stress are capable of modulating cellular activity and determining cellular fate, and work has begun to investigate the downstream effects of these phenomena in secreted EVs.^12^, ^13^, ^14^, ^15^, ^16^, ^17^, ^18^ Our group has reported on this previously, where MSCs subjected to flow‐derived shear stress in a perfusion bioreactor produced EVs that exerted a significantly more therapeutic angiogenic effect compared to both PBS and flask‐generated EVs in a mouse wound healing model.^19^

Here, using fabricated polydimethylsiloxane (PDMS) devices, we show the vascularization bioactivity of EVs from human bone marrow‐derived MSCs (BM‐MSCs) is affected by producer cell substrate stiffness, where seeding cells on softer substrates promotes the secretion of EVs with greater potency. We also show that the softer substrates improve BM‐MSC EV secretion and overall yield compared to conventional tissue culture flasks. Towards the goal of improving the scalability of MSC EV therapeutics, we further demonstrate that this mechanoresponsiveness is essentially conserved for EVs obtained from MSCs derived from induced pluripotent stem cells (iMSCs), which offer an essentially infinite source for consistent MSCs without donor variability.^20^ Overall, this work suggests that adjusting matrix stiffness is a simple, scalable, and cost‐effective way to significantly improve MSC EV potency for tissue repair applications. These results suggest a solution to some of the imminent bottlenecks (i.e., potency, donor variability) to clinical translation of MSC EV therapeutics, and the simplicity of this approach makes it easy to test and adapt for other mechanoresponsive cell/EV types.

## 2 METHODS

### 2.1 Cell culture

Human bone marrow‐derived mesenchymal stem cells (BM‐MSCs) were purchased from ATCC (PCS‐500‐012). Human induced pluripotent stem cell‐derived MSCs (iMSCs) were also purchased from ATCC (ACS‐7010). Both BM‐MSCs and iMSCs were cultured in Dulbecco's Modified Eagle's Medium (DMEM) (Corning; 10‐013‐CV) supplemented with 10% fetal bovine serum (FBS) (Cytiva; SH30910.03), 1% penicillin–streptomycin (P/S) (VWR; 45000‐652), and 1% MEM non‐essential amino acids (ThermoFisher Scientific; 11140050) in T‐175 tissue culture flasks. All BM‐MSCs were seeded at passage 4 in experiments.

Human umbilical vein endothelial cells (HUVECs) pooled from multiple donors were purchased from PromoCell (C‐12203) and cultured in endothelial growth medium (PromoCell; C‐22121) with 1% penicillin–streptomycin in T‐75 tissue culture flasks coated with 0.1% gelatin at 37°C for 1 h prior to seeding. During the experiments, HUVECs were maintained in endothelial basal medium (PromoCell; C‐22221) with 0.1% FBS and 1% P/S. HUVECs were used at passage 3 or 4 in all experiments.

RAW 264.7 macrophages were purchased from ATCC (TIB71) and cultured in DMEM (Corning; 10‐013‐CV) supplemented with 5% FBS (Cytiva; SH30910.03) and 1% penicillin–streptomycin (VWR; 45000‐652) in T‐175 tissue culture flasks.

### 2.2 Stiffness device fabrication, preparation, and seeding

Sylgard 184 polydimethylsiloxane (PDMS) (Krayden; DC2065622) was made by thoroughly mixing the silicone elastomer base and crosslinker reagent, both included within the kit, at different weight/weight ratios as specified (5:1, 10:1, 20:1, 33:1, and 50:1). PDMS was poured into each compartment of 100 mm × 15 mm compartmentalized petri dishes (Celltreat; 229684), placed in a desiccator for 20–30 min or until all air bubbles had been released, and cured for 1 h at 80°C. For all devices, the thickness of the PDMS layer was kept relatively constant by pouring the same amount of PDMS (2 g) was poured into each compartment.

For the mixing experiments, Sylgard 184 was prepared using a 10:1 base to curing agent ratio. Sylgard 527 PDMS (Krayden; DC1696742) was made by thoroughly mixing Part A and Part B at a 1:1 weight/weight ratio. The prepared Sylgard 184 and Sylgard 527 solutions were mixed at the specified weight/weight ratios (1:0, 10:1, 5:1, 1:1, 1:5, 1:10, and 0:1), and the final mixture was poured into each compartment of a 100 mm × 15 mm compartmentalized petri dishes (Celltreat; 229684). Dishes were placed in a desiccator for 20–30 min or until all air bubbles had been released and were cured for 20 h at 65°C. Once again, the thickness of the PDMS layer was kept constant.

Stiffness devices were cleaned by rinsing with 100% ethanol, deionized water, and 100% ethanol again, and drying with filtered air. Devices were then plasma treated for 3 min, and UV sterilized for 10 min. This was immediately followed by coating each PDMS surface with a 20 μg/mL type I rat tail collagen (Sigma‐Aldrich; C3867‐1VL) in 1× PBS solution and incubated at 37°C for at least 1 h, a surface coating procedure to make the innately hydrophobic PDMS surface hydrophilic, that has been well characterized prior.^21^, ^22^ T‐175 flasks were also coated with the collagen solution.

Upon seeding, collagen was removed and the surface was washed twice with 1× PBS. Either BM‐MSCs or iMSCs were seeded at a concentration of 5000 cells/mL in 2 mL of media per compartment and left to adhere overnight at 37°C before replacing with 4 mL of EV‐depleted media per compartment for conditioned media collection and subsequent EV isolation. 250,000 cells were also seeded in a collagen‐coated T‐175 flask as a control group.

### 2.3 EV isolation

Conditioned media was collected from the devices and flask and replaced with fresh EV‐depleted media every day for 3 days and subjected to three differential centrifugation spins at 1000 × *g* for 10 min, 2000 × *g* for 20 min, and 10,000 × *g* for 30 min, taking the supernatant from each step to be used in the next. The BM‐MSC EVs were isolated via ultracentrifugation, where the supernatant from the final spin was subjected to centrifugation at 118,000 × *g* for 2 h. The supernatant was then poured off and the pelleted EVs were resuspended in 1× PBS before transferring to a Nanosep 300 kDa MWCO spin column (VWR; 29,300‐636) and centrifuging at 8000 × *g* until PBS was removed. The EVs were washed twice in the same spin column and EVs on the filter were resuspended in 1× PBS and sterile filtered using a 0.2 μm syringe filter.

All iMSC EVs were isolated via tangential flow filtration (TFF), where the supernatant from the 30‐min centrifugation spin was passed through a 0.2 μm filter before isolating the EVs via TFF (Repligen; KrosFlo KR2i TFF system). Using a protocol adapted from Heinemann et al., the samples were concentrated down to 10–15 mL using a 100 kDa MWCO MidiKros mPES membrane (Repligen; D04‐E100‐05‐N) with six diafiltration steps and a transmembrane pressure of 5 psi.^23^ Samples were further concentrated using a 100 kDa centrifugal concentrator (Corning; 431486) and resuspended in 1× PBS.

### 2.4 EV characterization

EV concentration and size was quantified using nanoparticle tracking analysis (NTA) using a NanoSight LM10 (Malvern Panalytical Limited). Each sample was diluted to achieve 20–100 particles per frame for accurate measurement. Three 30 s videos were acquired, and camera levels and detection threshold were maintained between EV samples. Total protein concentration was measured using a bicinchoninic acid (BCA) assay per the manufacturer's protocol (G‐Biosciences; 785‐571).

Transmission electron microscopy (TEM) images of the EVs were obtained using a negative stain. EV samples were fixed using 4% electron microscopy‐grade paraformaldehyde (PFA) for 30 min at room temperature. A carbon film grid (Electron Microscopy Sciences; CF‐200‐Cu‐25) was placed on a droplet of the EV/PFA solution, and then washed by placing on a droplet of 1× PBS followed by 1% glutaraldehyde in PBS for 5 min. The grid was then washed using a droplet of MilliQ water, and then placed on a droplet of uranyl acetate replacement stain for 10 min (Electron Microscopy Sciences; 22405). Grids were allowed to completely dry before imaging using a JEM 2100 LaB6 TEM (JEOL USA Incorporated).

EV presence was confirmed using Western blotting. Equal amounts of protein or cell lysate (as specified within figures) were loaded into gels and transferred to a nitrocellulose membrane. Analysis for ALIX (Abcam; ab186429), CD63 (ThermoFisher Scientific; 25682‐1‐AP), TSG101 (Abcam; ab125011), calnexin (Cell Signaling Technology; 2679), and GAPDH (Cell Signaling Technology; 2118). All primary antibodies were incubated with the membrane overnight at 4°C and diluted at a 1:1000 dilution except for GAPDH, which was diluted at a 1:2000 dilution. The next day, goat anti‐rabbit IRDye 800CW (LI‐COR; 925‐32210) was incubated with the membrane at a 1:10,000 dilution for 1 h before imaging on an Odyssey CLx imager (LI‐COR).

### 2.5 Cell viability assay

The Cell Counting Kit 8 (WST/‐8/CCK8) from Abcam was used to measure cell viability (Abcam; ab228554). The specified PDMS variations were cast into 96‐well plates and incubated with collagen solution for at least 1 h at 37°C. Wells were washed twice with 1× PBS and seeded at a density of 3000 cells/well. A standard curve was also made by seeding cells in wells with no PDMS. The following day, the WST‐8 solution was prepared in the dark by diluting it 1:10 in EV‐depleted media. Media was aspirated out of the wells and replaced with 115 μL/well of the WST‐8 dilution. The plate was incubated for 4 h at 37°C. One hundred microliters from each well was then transferred into a new 96‐well plate and absorbance was measured via plate reader. Fresh EV‐depleted media was added back to the cells and the plate was put back into the incubator. This was repeated for the following 3–4 days.

### 2.6 Quantifying cell growth and proliferation on PDMS

Specified PDMS mixtures were cast into 24‐well plates (3 wells/day for 4 days) and incubated with collagen solution for at least 1 h at 37°C. Wells were washed twice with 1× PBS and seeded with 40,000 cells/well. The following day, media was replaced with EV‐depleted media. The next day, media was aspirated, cells were washed once with 1× PBS, and cells were trypsinized. Cells were then spun down at 270 × *g*, resuspended, and counted using a hemocytometer. This was repeated for the following 3 days.

### 2.7 Tube formation assay

To measure in vitro angiogenesis, 48‐well plates were coated with 60 μL of growth factor reduced Matrigel (Corning; 356230) and incubated at 37°C for 30 min. P4 HUVECs were then seeded at 35,000 cells/well with either endothelial growth media (PromoCell; C‐22121) with 1% penicillin–streptomycin (positive control), endothelial basal media (negative control), or endothelial basal media (PromoCell; C‐22221) with 0.1% FBS and 1% penicillin–streptomycin with 5E9 EVs/mL. 3–6 h later, tubes were imaged using a Nikon Eclipse Ti2 Microscope at 2× magnification and the number of fully formed loops were counted.

### 2.8 Gap closure assay

To assess endothelial cell proliferation and migration, 96‐well plates were coated with 0.1% gelatin and incubated at 37°C for 1 h. 15,000 P4 HUVECs/well were then seeded in endothelial growth media (PromoCell; C‐22121) with 1% penicillin–streptomycin. Once cells have formed a confluent monolayer (~24 h), a scratch was induced using a p200 pipette tip before washing with 1× PBS and serum‐starving for 1–3 h with endothelial basal media (PromoCell; C‐22221) with 0.1% FBS and 1% penicillin–streptomycin. Media was then replaced with either endothelial growth media (positive control), endothelial basal media (negative control), or 5E9 EVs/mL in endothelial basal media, and imaged. 16–20 h later, the cells were imaged in the same locations and the percent gap closure was quantified using ImageJ.

### 2.9 EV miRNA isolation and screening

EVs from iMSCs grown on Sylgard 527 PDMS devices and collagen‐coated flasks were isolated as described above. 1E11 EVs were lysed using QIAzol Lysis Reagent (Qiagen; 79306), RNA was isolated using RNeasy kits (Qiagen; 74104), and cDNA was synthesized using the miScript II RT kit (Qiagen; 218161) supplementing the kit‐provided reverse transcriptase mix with *E. coli* poly(A) polymerase (New England Biolabs; M0276L) and M‐MuLV reverse transcriptase (New England Biolabs; M0253L). Human MSC EV‐associated miRNAs were screened using the miProfile™ Human MSC exosome miRNA qPCR arrays from GeneCopoeia (GeneCopoeia; QM046‐B6) per the manufacturer's protocol. Quantitative polymerase chain reaction (qPCR) was performed using a QuantStudio 7 Flex qPCR system (ThermoFisher Scientific; 4485701) and data were analyzed using the delta–delta Ct method normalized to one of the provided housekeeping genes (SNORD47).

### 2.10 EV‐treated HUVEC mRNA isolation and screening

P4 HUVECs were seeded at 60,000 cells/well in a 24‐well plate. The following day, cells were treated with PBS (control), 5E9 particles/mL of EVs from iMSCs grown in collagen‐coated flasks, or 5E9 particles/mL of EVs from iMSCs grown on Sylgard 527 PDMS devices. 24 h later, cells were washed with 1× PBS and lysed using QIAzol Lysis Reagent (Qiagen; 79306). RNA was isolated using RNeasy kits (Qiagen; 74104), and cDNA was synthesized using the miScript II RT kit (Qiagen; 218161) supplementing the kit‐provided reverse transcriptase mix with *E. coli* poly(A) polymerase (New England Biolabs; M0276L) and M‐MuLV reverse transcriptase (New England Biolabs; M0253L). Angiogenesis‐related mRNAs were screened using the GeneQuery Human Angiogenesis qPCR Array Kit from ScienCell (ScienCell; GK017), per the manufacturer's protocol. qPCR was performed using a QuantStudio 7 Flex qPCR system (ThermoFisher Scientific; 4485701). mRNA array data were analyzed using the delta–delta Ct method using the average of the provided housekeeping genes provided. Results were validated by performing qPCR using PowerTrack SYBR Green Master Mix (ThermoFisher; A46109). Primer sequences used for validation are listed in Table S1. Data were analyzed using the delta–delta Ct method using GAPDH as a housekeeping gene. All qPCR results were normalized to the PBS‐treated HUVEC group and expressed as either the log2 of the fold change as seen in the heat map, or the fold change of mRNA as seen in the validation data.

### 2.11 Anti‐inflammatory assay

Seventy‐five thousand RAW264.7 mouse macrophages were seeded into each well of a 48‐well plate in DMEM with 5% FBS and 1% P/S. Twenty four hours later, cells were pre‐treated with either no treatment (positive control), 1 μg/mL dexamethasone (Sigma‐Aldrich; D4902‐25MG) (negative control), or 5E9 EVs/mL. The following day, media was replaced with 10 ng/mL lipopolysaccharide (LPS) (Sigma‐Aldrich; L4391‐1MG) in DMEM with 5% FBS and 1% P/S and left to incubate at 37°C for 4 h. The conditioned media was then collected and stored at −80°C until analysis. To determine the anti‐inflammatory effects of the EVs, pro‐inflammatory cytokines in the conditioned media were measured using the DuoSet ELISA kits for mouse IL‐6 (R&D Systems; DY406) and mouse TNF‐α (R&D Systems; DY410).

### 2.12 Statistical analysis

All data are presented as the mean ± standard deviation (SD). An ordinary one‐way ANOVA with Sidak's or Tukey's multiple comparisons tests was used to determine statistical significance (*p* < 0.05) between groups for the tube formation assays, gap closure assays, elastic moduli data, and EV production data. A two‐way ANOVA with Tukey's multiple comparison test was used to analyze cell viability and *t*‐tests were used to analyze the qPCR validation data. All statistical analyses were performed with Prism 9 (GraphPad Software). Statistical significance within figures is noted as ns *p* > 0.05, *p < 0.05, **p < 0.01, ***p < 0.0005, and ****p < 0.0001.

## 3 RESULTS

### 3.1 Substrate fabrication and EV characterization

**FIGURE 1:**
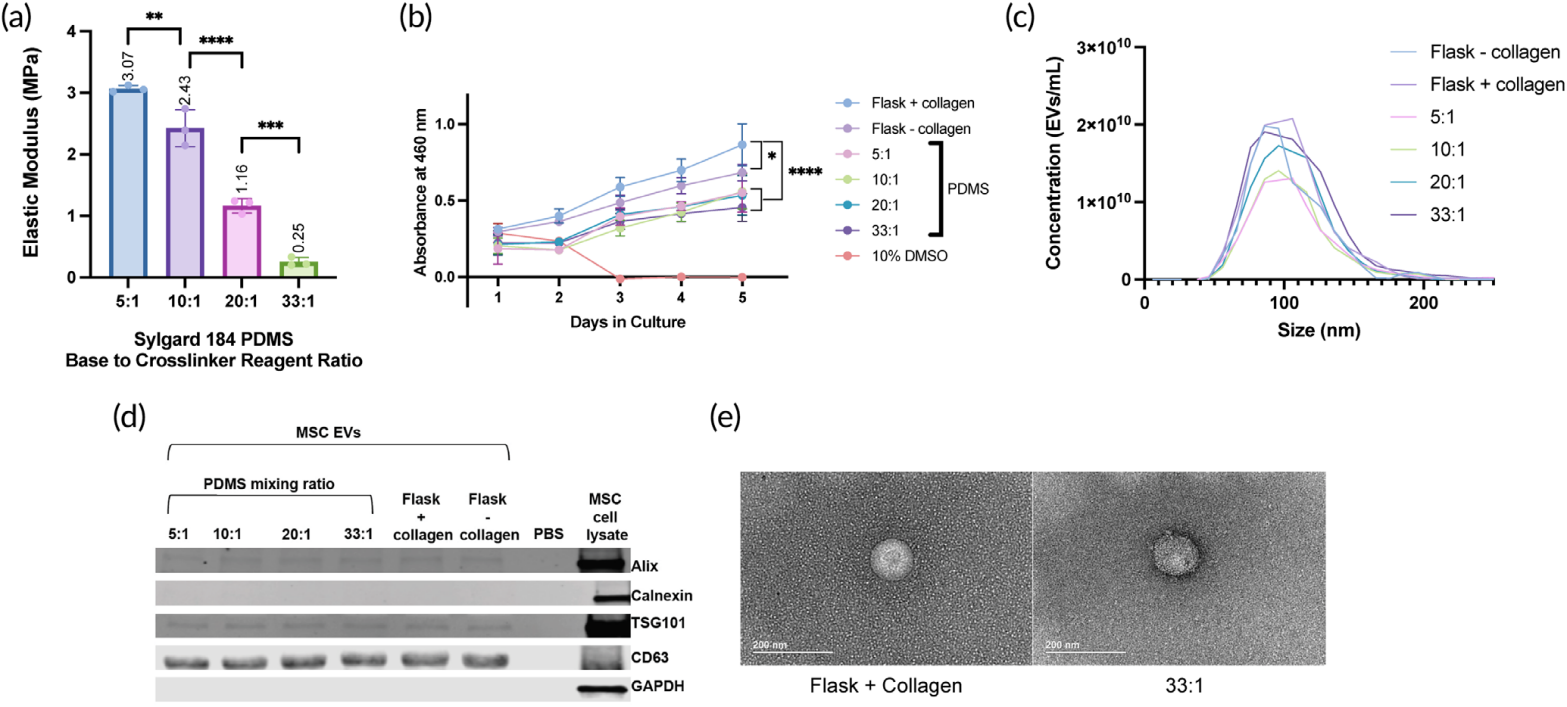
Substrate and BM‐MSC EV characterization. (a) Elastic moduli of substrates made with varying ratios of Sylgard 184 base to crosslinker reagents. All values expressed as mean ± SD (*n* = 3). (b) Absorbance values indicating cell viability as determined by CCK8 assay over 5 days. All values expressed as mean ± SD (*n* = 3). (c) Size distribution from nanoparticle tracking analysis of EVs isolated from BM‐MSCs seeded on Sylgard 184 PDMS substrates with differing base to crosslinker reagent ratios (*n* = 3). (d) Representative Western blot of BM‐MSC EVs from each of the Sylgard 184 PDMS substrates and the corresponding cell lysates for EV‐positive markers ALIX, TSG101, and CD63 and cellular markers Calnexin and GAPDH (15 μg/lane). (e) Representative TEM images of BM‐MSC EVs from the softest Sylgard 184 PDMS substrates and collagen‐coated flasks. Statistical significance was determined by ANOVA; **p < 0.01, ***p < 0.001, and ****p < 0.0001.

In order to understand how matrix stiffness affects MSC EV production and bioactivity, PDMS was used due to its high compatibility and tunable mechanical properties to create substrates of varying stiffnesses. A previous study demonstrated that altering the ratio of base to crosslinker reagent of Sylgard 184 PDMS allows for the creation of substrates with differing elastic moduli.^24^ Therefore, we initially fabricated a range of substrates using varying ratios of 184 PDMS base to crosslinker reagent. Mechanical testing using a Q800 Dynamic Mechanical Analyzer confirmed that substrates with elastic moduli of 250 ± 0.073 kPa, 1.16 ± 0.117 MPa, 2.43 ± 0.299 MPa, and 3.07 ± 0.054 MPa, were created using w/w ratios of 33:1, 20:1, 10:1, and 5:1, respectively (Figure 1a). A CCK8 assay was then used to confirm BM‐MSC viability over 5 days with 10% DMSO as a control, as the cells would not be grown on the substrates longer than 4 days (Figure 1b). We observed that although the cells on the PDMS substrates exhibited slightly lower absorbance values than those on the stiffer tissue culture polystyrene (TCPS) flasks, all groups except for the DMSO control trended upwards, indicating sustained viability. Additionally, despite slightly different starting absorbance values on day 1, there was no statistically significant difference between groups. We also confirmed that the elastic modulus was not affected by incubation with media (Figure S1a).

BM‐MSCs were seeded on each of these substrates with EV‐depleted media, and conditioned media was collected over a period of 3 days. Control EVs were from cells on both normal and collagen‐coated TCPS flasks. EVs were isolated via ultracentrifugation, and the size distribution and concentrations of each sample were determined using nanoparticle tracking analysis (NTA). The EVs from each group were all within the expected EV size distribution ranges, without any statistically significant differences in means or modes between the different groups (Figure 1c). EVs and cell lysates were analyzed via Western blotting to confirm EV identity. Here, all EV samples were positive for EV‐associated markers ALIX, TSG101, and CD63, while they were negative for cellular protein marker Calnexin (Figures 1d and S2). TEM images confirmed the size and spherical morphology of EVs from BM‐MSCs on both the stiffest (flask + collagen) and softest (33:1 184 PDMS) substrates, exhibiting no visual differences between EVs produced in either cell culture environment (Figure 1e).

### 3.2 Substrate stiffness influences BM‐MSC EV production and bioactivity

**FIGURE 2:**
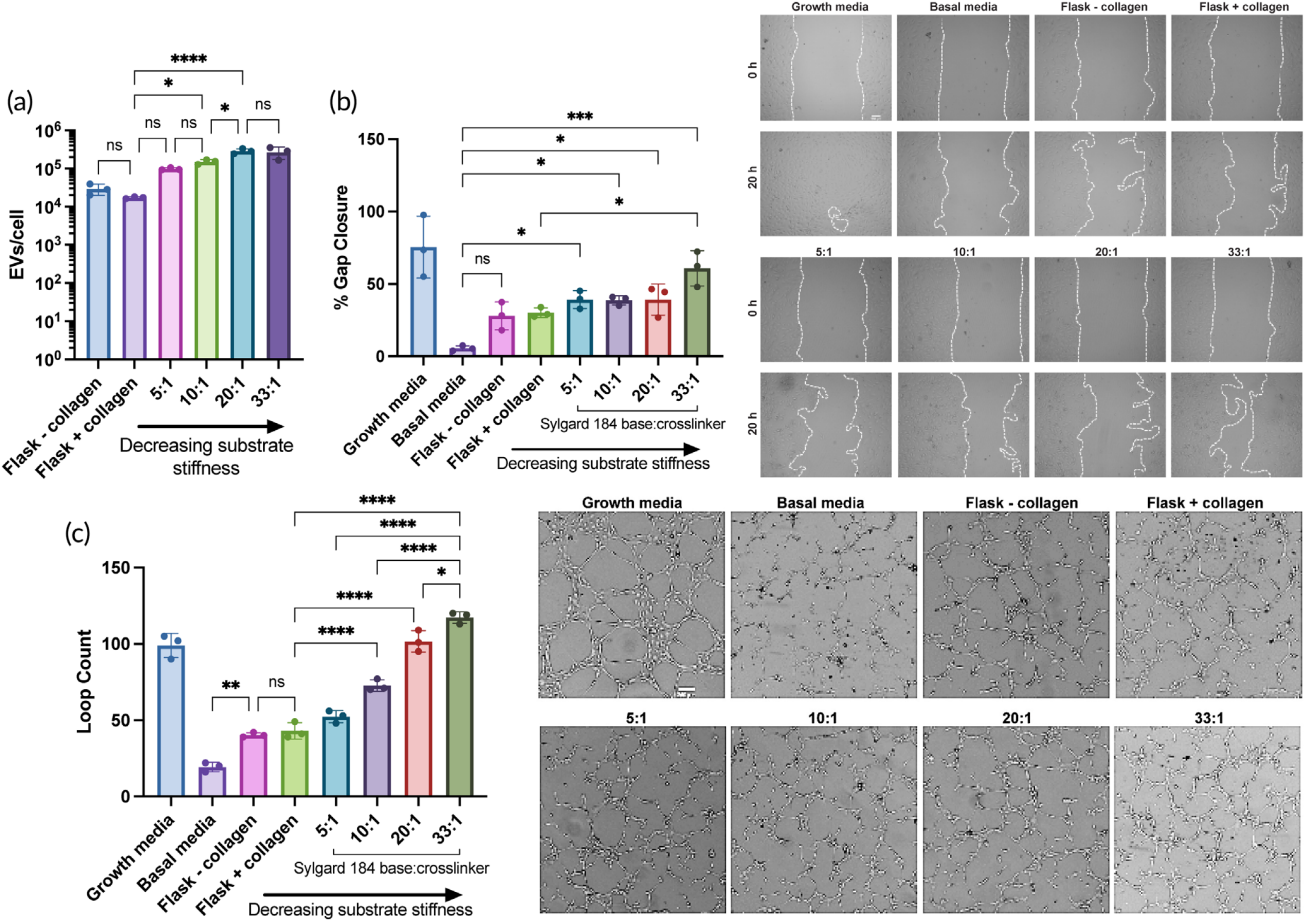
Substrate stiffness influences BM‐MSC EV production and bioactivity. (a) EV production as quantified by EVs per cell from BM‐MSCs seeded on Sylgard 184 PDMS substrates with different base‐to‐crosslinker ratios. EVs used for this data were from 1 day of collection and isolated and counted separately from the conditioned media from the other 2 days. After media collection, cells were trypsinized and counted (*n* = 3). (b) After a scratch was induced, HUVECs were treated with BM‐MSC EVs from the different substrates or growth or basal media, and percent gap closure after 20 h was evaluated via microscopy (*n* = 3). (c) HUVECs were resuspended in EV treatments or growth or basal endothelial media, seeded in Matrigel‐coated wells, and tube formation after 3–6 h was quantified by the number of loops that had formed (*n* = 3). All values expressed as mean ± SD. All data are representative of at least three independent experiments (*n* = 3). Statistical significance was determined by ANOVA; *p < 0.05, **p < 0.01, ***p < 0.001, and ****p < 0.0001.

Using EV concentration and cell count data, EV production was quantified as EV/cell to assess if substrate stiffness affects EV production. The 5:1, 10:1, 20:1, and 33:1 184 PDMS substrates resulted in EV production 5‐fold, 8‐fold, 16‐fold, and 15‐fold greater than the flask + collagen control, respectively (flask + collagen: 1.73E4, flask–collagen: 2.93E4, 5:1: 9.78E4, 10:1: 1.51E5, 20:1: 2.87E5, and 33:1: 2.69 E5) (Figure 2a).

We then sought to determine whether substrate stiffness had an effect on the angiogenic bioactivity of the generated EVs, since a common goal of regenerative medicine therapeutics is to promote vascularization and angiogenesis. To do so, we used two in vitro assays incorporating endothelial cells (human umbilical vein endothelial cells (HUVECs)) to model different stages of vascularization: a gap closure assay to measure cell proliferation and migration and a tube formation assay to model differentiation and re‐organization. 5E9 EVs/mL were used for these assays, as this dosing scheme has previously been shown to be effective in probing for differences in angiogenic activity of EVs from different culture conditions.^19^, ^25^ Importantly, we are able to see significant differences between the negative control (endothelial basal media) and the treatment groups.

Notably, EVs generated by the BM‐MSCs cultured on the softest (250 kPa, 33:1 184 PDMS) substrate caused significantly more cell migration and gap closure compared to the flask EVs and EVs from the other PDMS substrate groups (Figure 2b). This trend of increased angiogenic potential as a result of softer substrates was maintained in a tube formation assay, as EVs from the 33:1 184 PDMS substrate also significantly enhanced the number of loops compared to the rest of the EV groups (Figure 2c).

### 3.3 Mixing Sylgard 184 and Sylgard 527 PDMS provides a wider range of elastic moduli

**FIGURE 3:**
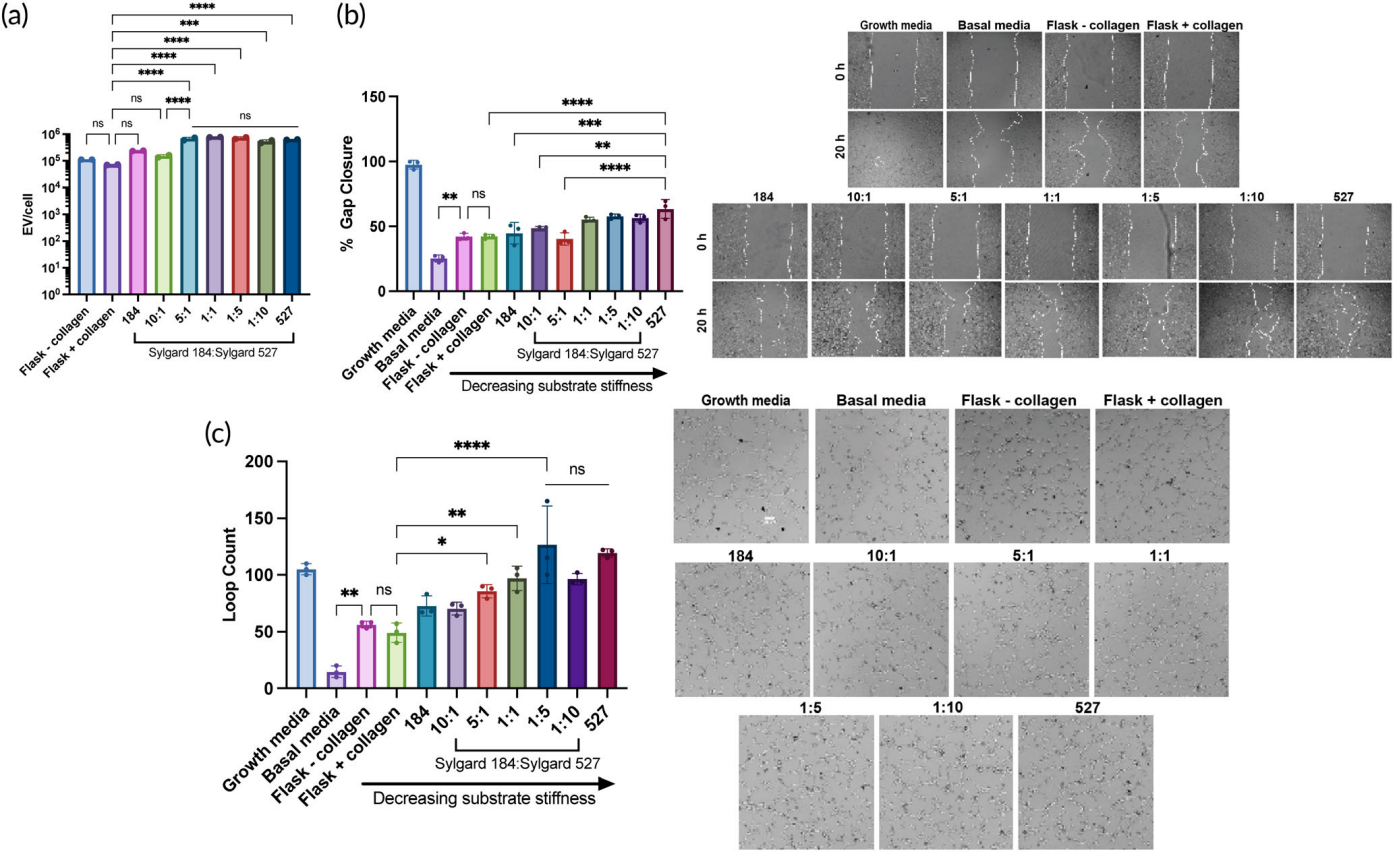
Softer 184:527 PDMS substrates improve the angiogenic bioactivity of BM‐MSC EVs. (a) EV production quantified as EV per cell from BM‐MSCs seeded on each substrate made with different ratios of Sylgard 184 and Sylgard 527 (*n* = 2). EVs used for this data were from 1 day of collection and isolated and counted separately from the conditioned media from the other 2 days. After media collection, cells were trypsinized and counted. (b) After a scratch was induced, HUVECs were treated with BM‐MSC EVs from the different substrates or growth or basal media, and percent gap closure after 20 h was evaluated via microscopy (*n* = 3). (c) HUVECs were resuspended in the different EV treatments or growth or basal endothelial basal media, and tube formation after 3–6 h was quantified by the number of loops that had formed (*n* = 3). All values expressed as mean ± SD. Statistical significance was determined by ANOVA; *p < 0.05, **p < 0.01, and ****p < 0.0001.

To examine a wider range of elastic moduli, we mixed Sylgard 184 and Sylgard 527, which is reported to yield PDMS substrates with a range of elastic moduli between ~3 kPa and ~^26^ We confirmed that we could also achieve this range by mixing Sylgard 184, which was prepared at a 10:1 base to crosslinker reagent ratio per the manufacturer's instructions, and Sylgard 527 at w/w ratios of 1:0 (pure 184), 10:1, 5:1, 1:1, 1:5, 1:10, and 0:1 (pure 527) (Figures S1b and S3a). We also showed that BM‐MSCs grown on these substrates remained viable and generated EVs similar to those from the prior characterized PDMS devices (Figure S3b,c). Although the difference in EV production per cell was not significant between the 5:1, 1:1, 1:5, 1:10, and 527 groups, they all showed increased production when compared to the 184 and 10:1 groups, as well as the flask controls (Figure 3a). When looking at angiogenic bioactivity, treatment with 5E9 EVs/mL from the softer substrates, especially the 527 PDMS, induced significantly higher levels of gap closure compared to the softer PDMS substrates (184, 10:1, 5:1) (Figure 3b), continuing the trend seen with the 184 PDMS substrate variations. Furthermore, treatment with 5E9 EVs/mL from the softer substrates, particularly the 139 kPa 1:5, 54 kPa 1:10, and 3 kPa 527 devices, resulted in significantly greater tube formation than EVs from the stiffer PDMS devices and flasks (Figure 3c), suggesting that the substate stiffness‐dependent change in angiogenic activity continues past the stiffnesses that were achieved when using only Sylgard 184.

### 3.4 iMSC EVs are mechanoresponsive and comparable pro‐angiogenic treatments to BM‐MSC EVs

Donor‐sourced MSCs have inherently variable properties based on the characteristics of the donor. Our group has previously demonstrated the significant variability in both the angiogenic and anti‐inflammatory bioactivity of BM‐MSC EVs from different donors.^19^ Additionally, prior work from our group has shown a decrease in functional activity of BM‐MSC EVs, specifically in their angiogenic activity, after passage 4, which substantially limits their expansion capacity and makes donor MSCs unideal for clinically translatable therapeutics.^27^ Therefore, we aimed to assess whether induced pluripotent stem cell‐derived MSCs (iMSCs) are a suitable alternative to BM‐MSCs in the context of enhancing their pro‐angiogenic effect via mechanical regulation.

**FIGURE 4:**
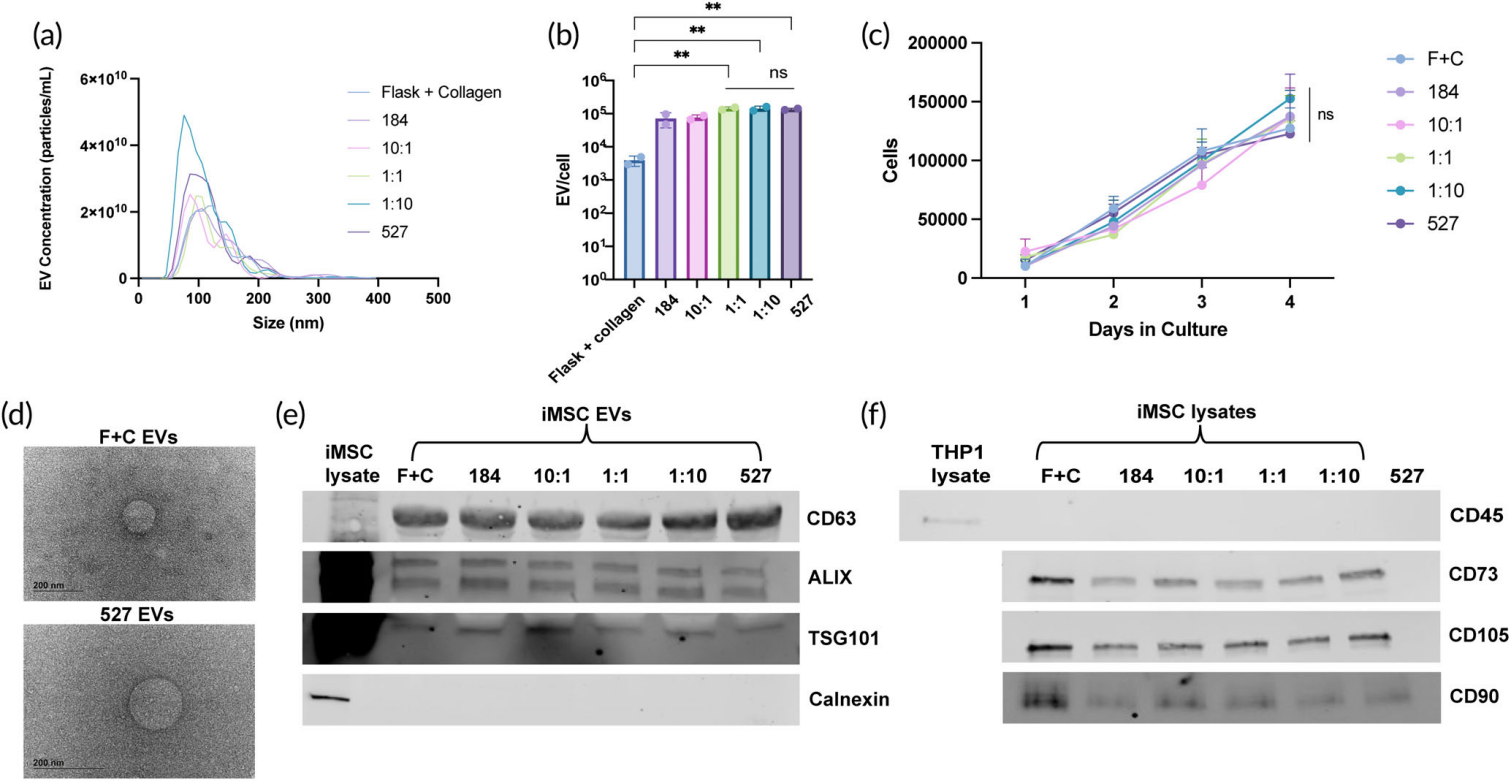
(a) EV production as quantified by EV per cell by EVs from iMSCs on different PDMS substrates. (b) EV size and concentration distribution from iMSCs cultured on different PDMS substrates as determined by nanoparticle tracking analysis. (c) iMSC proliferation/viability on PDMS substrates as measured by cell counting over 4 days. (d) Representative TEM images of F + C EVs and 527 EVs confirming morphology. (e) Western blot of EV markers CD63, ALIX, and TSG101, and EV‐negative marker, calnexin, on EVs from each PDMS substrate (12 μg/lane). (f) Western blot of MSC markers CD73, CD105, and CD90 and negative marker CD45 on iMSC lysate from each PDMS substrate. THP1 cell lysate was used as a positive control for CD45. (5 μg/lane). All values expressed as mean ± SD. Statistical significance was determined by ANOVA; **p < 0.01.

iMSCs were seeded on the mixed PDMS substrates as described before, using only the 184, 10:1 1:1, 1:10, and 527 groups, as the differences in the elastic moduli were pronounced enough to still observe a clear trend in angiogenic activity. The treatment control substrate was reduced to just collagen‐coated flasks, as the difference in the flask + collagen and flask–collagen groups were consistently not significant. EVs were isolated and characterized with NTA to determine size distribution and concentrations of the samples, which showed the expected EV diameters (100–200 nm) (Figure 4a). We then quantified iMSC EV production per cell and found that the three softest substrate groups (1:1, 1:10, and 527) resulted in a statistically significant increase in EV production when compared to flask culture (Figure 4b). These results were also similar to those produced with BM‐MSC EVs. We also ensured that the iMSCs were viable and be maintained on PDMS as the BM‐MSCs were (Figure 4c). An upward trend in cell proliferation was confirmed over the course of 4 days since the iMSCs are not in culture longer than 4 days during the EV isolation process. TEM images confirmed the spherical EV morphology as expected from iMSCs on both TCPS and the 527 PDMS (Figure 4d), and Western blot confirmed EV identity as EVs from all substrates were positive for EV markers CD63, ALIX, TSG101, and negative for cellular marker calnexin (Figures 4e and S4). Finally, we determined that MSC identity was maintained after the EV collection process by Western blots for the presence of CD90, CD73, and CD105, and the absence of CD45 (Figures 4f and S5).

**FIGURE 5:**
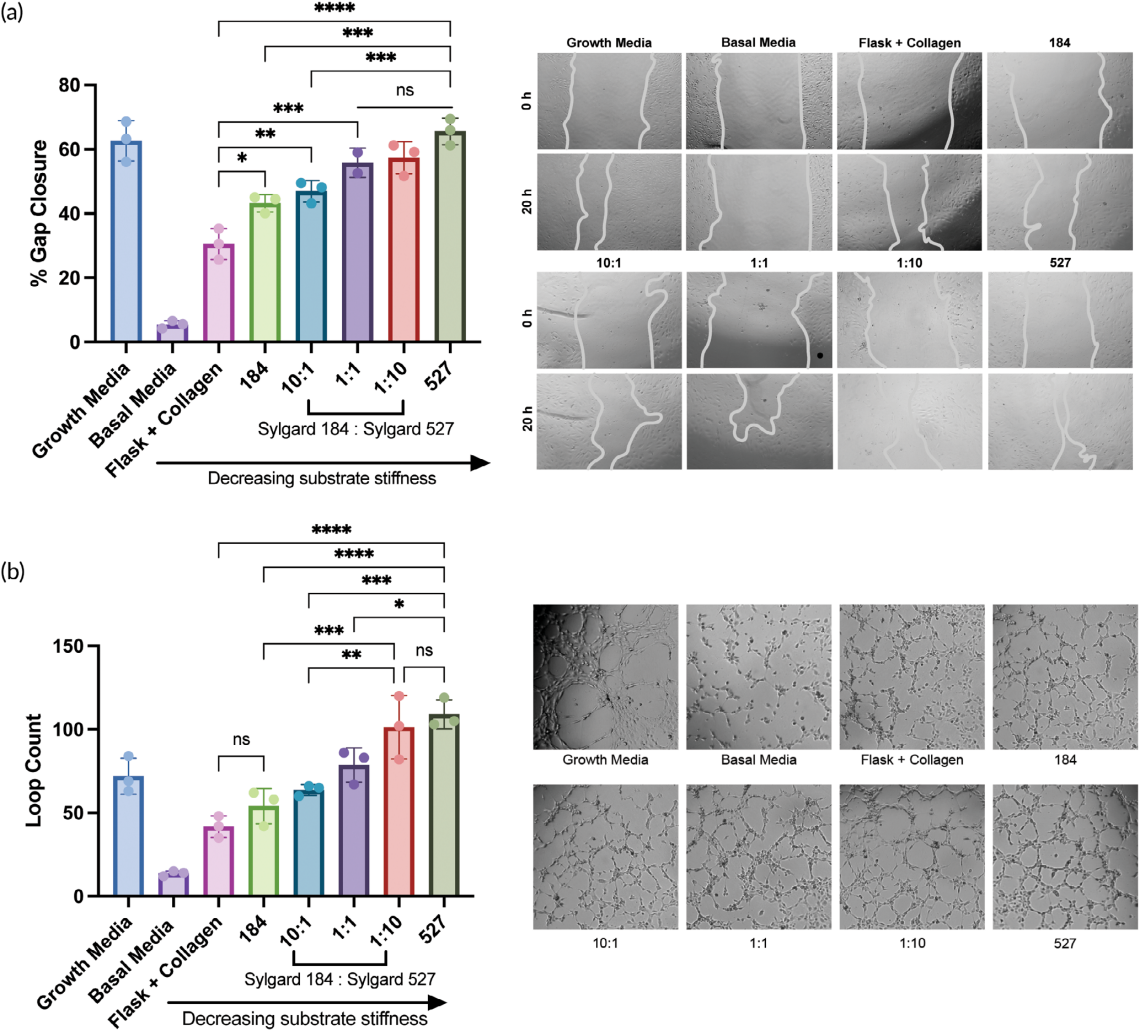
Substrate stiffness affects the pro‐angiogenic effect of iMSC EVs comparably to BM‐MSC EVs. (a) EVs isolated from iMSCs on different 184:527 PDMS substrates were used to treat HUVECs after a scratch had been induced, and percent gap closure after 20 h was evaluated via microscopy. (b) HUVECs were resuspended with the same EV groups and seeded, and tube formation after 3–6 h was quantified by the number of loops that had formed. All values expressed as mean ± SD. All data are representative of at least three independent experiments (*n* = 3). Statistical significance was determined by ANOVA; *p < 0.05, **p < 0.01, ***p < 0.001, and ****p < 0.0001.

Upon investigating iMSCs' mechanoresponsiveness and their EV bioactivity, we observed that like BM‐MSC EVs, treatment with 5E9 EVs/mL iMSC EVs caused significantly more gap closure compared to the endothelial basal media control (Figure 5a). The iMSCs also exhibited mechanoresponsiveness, as we show a pronounced substrate stiffness‐dependent effect on bioactivity where the softest substrate induced the most gap closure. This trend was reflected in a tube formation assay as well, where 5E9 EVs/mL iMSC EVs enhanced the number of loops formed by the HUVECs, with a significantly more potent angiogenic effect as substrate stiffness decreased (Figure 5b). It should be noted that the mechanoresponsiveness and trend in substrate stiffness‐dependent change in bioactivity also continued past the elastic moduli of the softest 184 PDMS substrate (33:1, 250 kPa) since the elastic moduli of the 1:1, 1:10, and pure 527 substrates were 139, 54, and 3 kPa, respectively. Moreover, since wound healing and tissue repair processes include a shift from a pro‐inflammatory state to an anti‐inflammatory state, we also confirmed that substrate stiffness does not significantly affect the anti‐inflammatory activity of the iMSC EVs upon looking at levels of pro‐inflammatory TNF‐α and IL‐6 (Figure S6).

### 3.5 Investigating potential mechanistic changes as a result of decreased substrate stiffness

**FIGURE 6:**
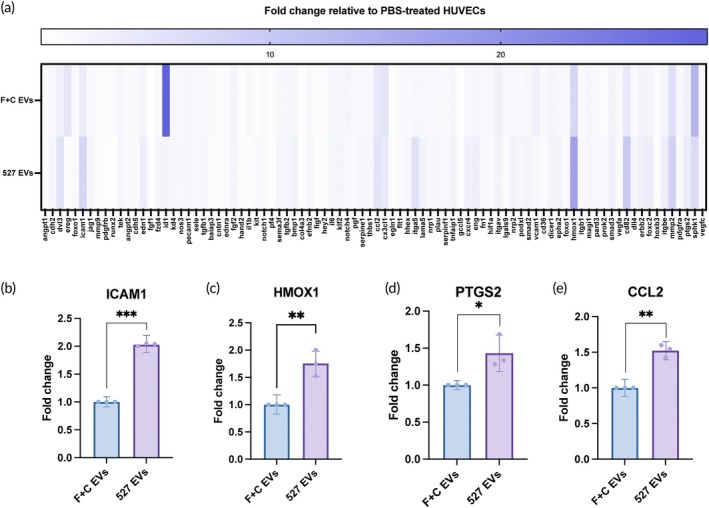
HUVECs treated with 5E9 EVs/mL of soft substrate‐generated iMSC EVs result in the upregulation of certain angiogenesis‐related mRNAs. (a) mRNA array probing for expression of angiogenesis‐related genes in flask‐ and soft substrate (527 PDMS)‐generated EV‐treated HUVECs normalized to PBS‐treated HUVECs (*n* = 1). (b)–(e) Confirmation of upregulation of genes (ICAM1, HMOX1, PTGS2, and CCL2) selected from the initial array by independent qPCR experiments (*n* = 3). Statistical significance was determined by *t*‐test; *p < 0.05, **p < 0.01, and ***p < 0.001.

Given that the EVs from cells on softer substrates exhibited a marked enhancement in pro‐angiogenic activity, it is likely that there are genetic changes occurring that contribute to this effect. We first used an mRNA array to screen 88 angiogenesis‐related genes comparing the lysate of HUVECs treated with PBS, HUVECs treated with soft substrate (527 PDMS)‐generated EVs, and HUVECs treated with flask‐generated EVs. When treated with the soft substrate‐generated EVs, 25 genes were upregulated more than 2‐fold compared to the PBS‐treated cells (Figure 6a). We then proceeded to confirm the differential expression that was suggested in the array for the top candidates (i.e., the genes with the highest fold changes) that were also upregulated when compared to the flask EV‐treated HUVECs and determined that four were most consistently upregulated in correlation with treatment by the soft substrate‐generated EVs (ICAM1, HMOX1, PTGS2, and CCL2) (Figure 6b–e).

**FIGURE 7:**
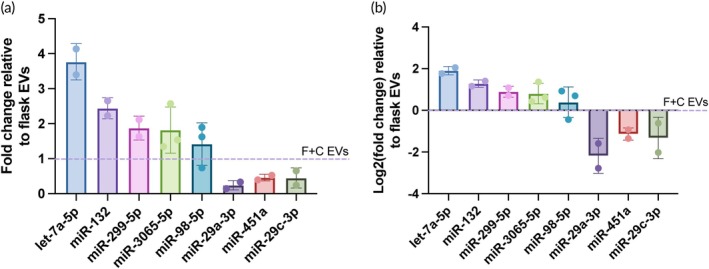
MiRNA qPCR array data of differentially expressed MSC EV‐associated miRNAs in flask vs. soft substrate‐generated EVs. (a) Fold change of miRNAs within EVs from iMSCs seeded on 527 PDMS normalized to the miRNA levels within EVs from iMSCs seeded on collagen‐coated flasks. (b) The same data represented as the log2 of the fold change, again normalized to the miRNA levels within EVs from iMSCs seeded on collagen‐coated flasks. All data are representative of at least three independent experiments (*n* = 3).

We also investigated changes in the miRNA profile of the EVs themselves based on the stiffness of the substrates the cells were cultured on. In contrast to the mRNA qPCR array, a miRNA array on the EV‐associated RNA provided an alternative approach to explore molecular changes within the EVs themselves that are associated with culturing iMSCs on softer substrates. Thus, a miRNA array screening for human MSC EV‐associated miRNAs was used to evaluate the content of EVs from the 527 PDMS substrates and collagen‐coated flask substrates. While there was variation between each biological replicate, particularly with respect to which miRNAs were successfully amplified given the low miRNA levels within EV samples and being limited to a single technical replicate, we observed that two miRNAs were upregulated in the soft substrate‐generated EVs compared to the flask EVs more than 2‐fold across at least two of the biological replicates performed (let‐7a‐5p, miR‐132) (Figure 7). Three others were upregulated across at least two of the replicates more than 1.5‐fold (miR‐299‐5p, miR‐3065‐5p, and miR‐98‐5p). We also observed two miRNAs that were downregulated more than 2‐fold across at least two of the replicates (miR‐299‐3p, miR‐451a) and one downregulated more than 1.5‐fold (miR‐29c‐3p). It is important to note that while the data from both the mRNA array (Figure 6) and miRNA array (Figure 7) could be used as a baseline to pursue further mechanistic studies in the future, here they serve only to show association with softer cell culture substrates.

## 4 DISCUSSION

MSC EVs remain a promising therapeutic for regenerative medicine applications, comparable or superior to cell therapies in some applications.^28^, ^29^ Accordingly, there has been a rise in the number of clinical trials evaluating MSC EVs for a variety of therapeutic applications such as graft‐versus‐host disease, cardiac repair, and wound healing.^30^, ^31^, ^32^ In particular, the therapeutic application of MSC EVs for tissue repair and angiogenesis‐related processes is well‐recognized and widely studied. However, FDA‐approval and ultimate clinical translation of MSC EVs has been hindered by a multitude of barriers including therapeutic potency, scalability, and donor variability, all of which need to be addressed to successfully implement EV therapies in the clinic.^4^, ^5^ Common approaches to improve potency often fall into categories of either endogenous or exogenous loading of therapeutic RNAs, proteins, or drugs, which can be effective but are also associated with high costs, EV degradation, and increased manufacturing and regulatory burden.^33^ Additionally, it is well established by others and work from our lab that variability among donor MSCs is reflected in the function and efficacy of their EVs and a crucial obstacle to overcome.^19^, ^34^, ^35^ Our group has also shown that there is a significant drop‐off in the bioactivity of donor‐derived BM‐MSC EVs after passage 4, further limiting the scale‐up capabilities of donor MSCs.^27^ Therefore, there is a clear need for alternative ways to improve the therapeutic potency of MSC EVs, as well as identifying an alternative MSC source for EV production to eventually achieve clinical translation.

Mechanical regulation of cells and the effects of biophysical stimuli such as fluid shear stress, stretch and compression, and even substrate stiffness have been well studied in the context of the cellular response, and their effects continue to be elucidated.^12^, ^13^, ^14^, ^18^, ^36^, ^37^ With MSCs specifically, it has also been established that matrix stiffness can direct differentiation.^26^, ^38^, ^39^ Notably, there is evidence that EVs also respond to mechanical stimuli. For instance, Guo et al. reported that the mechanical cues of flow and cyclic stretch improved stem cell EV bioactivity with respect to promoting axonal sprouting.^40^ Subjecting producer cells to fluid shear stress is also commonly reported by many groups, including ours, to improve EV production.^19^, ^41^, ^42^ Mechanical compression or squeezing has also been shown to promote MSC EV generation.^43^ Here, we focused on elucidating the specific impact of substrate stiffness on MSC EV production and bioactivity. Given that BM‐MSCs and BM‐MSC EVs have been widely studied with respect to their inherently pro‐angiogenic and vasculogenic effects for a variety of tissue regeneration and vascularization applications such as chronic wound healing and cardiac repair, we initially started this work using BM‐MSCs as the EV producer cells.

By changing the base to crosslinker reagent ratios of Sylgard 184 PDMS to create substrates with stiffnesses ranging from 250 kPa to 3 MPa (Figure 1), we showed that seeding BM‐MSCs on softer substrates enhanced EV production per cell (Figure 2a). This is consistent with other reports evaluating the effects of substrate stiffness on MSC EV secretion, where Lenzini et al. showed that 3 kPa alginate hydrogels coated with cell adhesion peptides were able to improve EV secretion from BM‐MSCs 10‐fold compared to cells seeded on flasks.^15^ Interestingly, there seems to be an opposite trend in the context of tumors where matrix stiffening promotes EV secretion to contribute to growth and metastasis, although when comparing stiffnesses, the range is much smaller on the scale of ~0.5–25 kPa.^44^, ^45^ In the case of therapeutic, regenerative EVs, an increase in production by simply changing cell substrates is favorable for the goal of clinical translation, as greater production per batch will allow for fewer batches per dose. Towards the same goal of improving the potential for clinical translation of pro‐regenerative MSC EVs, we demonstrated an increase in the angiogenic activity of BM‐MSC EVs from cells cultured on a 250 kPa PDMS substrate (33:1) compared to the stiffer PDMS variations or flasks (Figure 2b,c). However, out of all the PDMS stiffnesses tested here, this increase was mainly seen with only the softest substrate. Thus, we aimed to increase the range of elastic moduli tested by mixing a stiffer PDMS, Sylgard 184 (made at a 10:1 base to crosslinker reagent ratio), and a softer PDMS, Sylgard 527, at different ratios. Substrates with stiffnesses ranging from 3 kPa to 2 MPa were achieved, extending the range that could be tested past what was feasible with only the Sylgard 184 (S3A). While we observed a stiffness‐dependent enhancement in EV production per cell between the 184, 10:1, and 5:1 groups, there was no additional change in EV secretion with the 1:1, 1:5, 1:10, and 527 groups (Figure 3a). This might be explained by the fact that the cells may have reached a maximum level of EVs they can secrete during one collection period, regardless of stiffness level. As we expected, the trend of increasing BM‐MSC EV bioactivity as substrate stiffness decreased was maintained, although the tube formation by the HUVECs treated with EVs from the three softest substrates was not significantly different (Figure 3b,c). However, while the elastic moduli of the 1:5, 1:10, and 527 substrates were significantly softer than the stiffer 184, 10:1, 5:1, and 1:1 substrates and good representatives of a soft matrix, their elastic moduli were not significantly different from each other (Figure S3a), which may explain these results. Regardless, the ability to enhance the pro‐regenerative function of BM‐MSC EVs by tuning substrate stiffness is a promising and potentially scalable approach.

In the interest of further improving scalability, we chose to also investigate an alternative MSC source for generation of MSC EVs with enhanced therapeutic efficacy. Donor variability is a well‐established challenge in the field of stem cell therapies, and numerous data, including from our group, have demonstrated differences in bioactivity between BM‐MSCs from different donors.^19^, ^34^, ^35^ We have also observed a dampened effect in functionality at higher passages, limiting their use and practicality in scaled up processes.^27^ Induced pluripotent stem cells (iPSCs) are self‐renewing and can be differentiated into MSCs.^46^ Thus, these iMSCs offer the potential for indefinite expansion in culture, making them an ideal candidate for consistent, alternative cell lines to donor MSCs that could be scaled up more successfully. Additionally, in the interest of improving scale‐up potential, we began using TFF, a separation method offering superior yield, purity, reproducibility, and scalability when compared to ultracentrifugation to isolate all EVs moving forward.^47^ Using iMSCs, we were able to confirm their EVs' vascularization bioactivity and mechanoresponsiveness as we did with BM‐MSC EVs and saw that their ability to induce gap closure and tube formation increased as the matrix stiffness decreased (Figure 5). These results were expected given that the cell source was still phenotypically iMSCs, albeit from a different tissue source. Gultian et al. performed a study that bolsters these results as well, where comparing iMSCs and BM‐MSCs showed that iMSCs exhibited equal or higher and less variable levels of stiffness‐driven nuclear localization of yes‐associated protein (YAP), as well as phosphorylated focal adhesion kinase, both of which are essential components of mechanotransduction pathways.^48^ Together with our data, this further promotes iMSCs as an equivalent alternative to BM‐MSCs as producers of EVs with improved potency via mechanical regulation.

Finally, we began looking into any genetic changes that might have occurred as a result of the different substrate stiffnesses. The goal of these experiments was to simply identify genes and miRNAs that show increased or decreased expression based on softer substrates and demonstrate association between such molecules and substrate stiffness. We first evaluated potential differential expression of angiogenesis‐related genes in HUVECs treated with soft or stiff substrate‐generated EVs through an mRNA array (Figure 6a). While a qPCR array provides only an overarching screen with low statistical power, we sought to provide more confirmation by performing individual qPCR experiments where, out of the eight angiogenesis‐associated genes that were selected from the array, four (ICAM1, HMOX1, PTGS2, and CCL2) were most consistently differentially expressed (Figure 6b–e). However, for a more accurate representation of upregulated mRNAs to probe further for more in‐depth mechanistic studies, it would be beneficial to perform more replicates of the initial qPCR array in the future. Nevertheless, these data provide insight into the effects that the EVs from different substrates have on HUVECs and provides a platform for future work to elucidate mechanism of the increased angiogenesis.

EV‐associated miRNA content was also assessed, as miRNAs are common effector molecules that are abundant in EVs and EV miRNA content is known to be regulated by mechanical stimuli.^49^, ^50^, ^51^, ^52^ Again, the statistical power of a qPCR array is low given that there is only one replicate per miRNA being evaluated. Additionally, the number of miRNAs within a qPCR array that were able to be successfully amplified was limited due to naturally low levels of endogenous miRNA within EVs. Thus, three biological replicates were performed, where let‐7a‐5p and miR‐132 were upregulated more than 2‐fold and miR‐299‐5p, miR‐3065‐5p, and miR‐98‐5p were upregulated more than 1.5‐fold in the soft substrate‐generated EVs relative to the flask‐generated EVs across at least 2 of the replicates (Figure 7). Of note, all of these miRNAs except for miR‐98‐5p have been documented to be associated with the promotion of cell proliferation and migration and/or angiogenesis in various conditions, although for some, their role seems to depend on the physiological condition.^53^, ^54^, ^55^, ^56^ Interestingly, miR‐98‐5p seems to mainly be associated with inhibitory effects in the context of angiogenesis.^57^, ^58^, ^59^ However, it has also been shown to exert anti‐inflammatory effects which may explain its presence,^60^, ^61^ especially its more minimal upregulation in soft substrate‐generated EVs relative to flask EVs since both are anti‐inflammatory without significant differences in potency (Figure S6). We also showed the downregulation of miR‐29a‐3p and miR‐451a more than 2‐fold, and miR‐29c‐3p more than 1.5‐fold in the soft substrate‐generated EVs (Figure 7). These results are consistent with other reports that have shown that these miRNAs inhibit or suppress angiogenesis and cell proliferation and migration.^62^, ^63^, ^64^ Notably, Deng et al. showed that miR‐29a‐3p overexpression significantly decreased the expression levels of ICAM1 in vitro in HUVECs and in vivo in the aortic endothelium of mice in the context of atherosclerosis.^65^ This aligns with our data here where miR‐29a‐3p was downregulated in the soft substrate‐generated EVs and ICAM1 was upregulated in the HUVECs treated by the same EVs compared to in HUVECs treated by flask EVs, further supporting a potential relationship between the two that could be explored in the future. In future work, these identified miRNAs could be used as a baseline for more in‐depth mechanistic studies and functional/pathway analysis. For instance, the data here in combination with the angiogenesis‐related mRNAs may also help narrow down specific mechanisms to further pursue since we have presented both potential effector miRNAs in the EVs and resulting changes in cells treated with the EVs.

## 5 CONCLUSIONS

In conclusion, these studies reveal that the mechanical cue of substrate stiffness can be tuned to increase yield and enhance bioactivity of MSC EVs. The data also establish the mechanoresponsiveness and general efficacy of iMSC EVs as an alternative to BM‐MSC EVs uninhibited by reliance on multiple donors for mass production and the associated inherent variability.

## AUTHOR CONTRIBUTIONS

Emily H. Powsner (Conceptualization, Investigation, Writing—original draft, Methodology, Validation, Visualization, Writing—review & editing); Stephanie M. Kronstadt (Conceptualization, Investigation, Writing—review & editing, Methodology, Validation); Kristin Nikolov (Investigation, Validation); Amaya Aranda (Investigation, Validation); Steven M. Jay (Conceptualization, Funding acquisition, Writing—original draft, Writing—review & editing, Supervision, Project administration).

## CONFLICT OF INTEREST STATEMENT

The authors declare no conflicts of interest.

## Supporting information


**FIGURE S1:** Incubation in media does not significantly affect the elastic modulus of the PDMS substrates. Elastic modulus of the (a) Sylgard 184 PDMS devices and (b) mixed Sylgard 184 and Sylgard 527 PDMS devices before and after being incubated in DMEM a 37ºC for 4 days. All values expressed as mean ± SD. All data are representative of at least two independent experiments (*n* = 2). Statistical significance was determined by *t*‐test; ns, not significant.
**FIGURE S2:** Full EV marker Western blot membranes after imaging (black and white) for Figure 1d.
**FIGURE S3:** Mixed Sylgard 184 and Sylgard 527 PDMS substrate characterization. (a) Elastic moduli of stiffness devices made with varying ratios of Sylgard 184 and Sylgard 527 PDMS. Values expressed as mean ± SD (*n* = 4). (b) Absorbance values from a CCK8 assay indicating cell viability over 4 days. Values expressed as mean ± SEM (*n* = 2). (c) Size distribution from NTA of EVs from BM‐MSCs seeded on flasks or each substrate made with different ratios of Sylgard 184 and Sylgard 527. Statistical significance was determined by ANOVA; *p < 0.05, ***p < 0.001, ****p < 0.0001.
**FIGURE S4:** Full EV marker Western blot membranes after imaging (black and white) and ponceau‐stained after protein transfer step to confirm protein loading for Figure 4e.
**FIGURE S5:** Full MSC marker Western blot membranes after imaging (black and white) and ponceau‐stained after protein transfer step to confirm protein loading for Figure 4f.
**FIGURE S6:** Substrate stiffness does not have a significant effect on the anti‐inflammatory effects of iMSC EVs. Levels of pro‐inflammatory cytokines (a) IL‐6 and (b) TNF‐α in conditioned media of RAW264.7 cells treated with 5E9 EVs/mL in an LPS‐stimulated mouse macrophage inflammatory assay, quantified by an ELISA. Dexamethasone (dex) served as a positive control to reduce inflammation. All values expressed as mean ± SD. Data are representative of at least three independent experiments (*n* = 3). Statistical significance was determined by ANOVA; ns, not significant, **p < 0.01, ***p < 0.001, and ****p < 0.0001.
**TABLE S1:** qPCR primers used for validation of angiogenesis‐related genes from the mRNA array.

## Data Availability

The data that support the findings of this study are available from the corresponding author upon reasonable request.
