# Supplementary material for: Mesenchymal stem cell extracellular vesicle vascularization bioactivity and production yield are responsive to cell culture substrate stiffness
Source: Bioeng Transl Med. 2025 Jan 7;10(3):e10743. doi: 10.1002/btm2.10743 (PMC12079338; doi:10.1002/btm2.10743)
Supplement: Supplementary file 1 — FIGURE S1: Incubation in media does not significantly affect the elastic modulus of the PDMS substrates. Elastic modulus of the (a) Sylgard 184 PDMS devices and (b) mixed Sylgard 184 and Sylgard 527 PDMS devices before and after being incubated in DMEM a 37ºC for 4 days. All values expressed as mean ± SD. All data are representative of at least two independent experiments (n = 2). Statistical significance was determined by t‐test; ns, not significant. FIGURE S2: Full EV marker Western blot membranes after imaging (black and white) for Figure 1d. FIGURE S3: Mixed Sylgard 184 and Sylgard 527 PDMS substrate characterization. (a) Elastic moduli of stiffness devices made with varying ratios of Sylgard 184 and Sylgard 527 PDMS. Values expressed as mean ± SD (n = 4). (b) Absorbance values from a CCK8 assay indicating cell viability over 4 days. Values expressed as mean ± SEM (n = 2). (c) Size distribution from NTA of EVs from BM‐MSCs seeded on flasks or each substrate made with different ratios of Sylgard 184 and Sylgard 527. Statistical significance was determined by ANOVA; *p < 0.05, ***p < 0.001, ****p < 0.0001. FIGURE S4: Full EV marker Western blot membranes after imaging (black and white) and ponceau‐stained after protein transfer step to confirm protein loading for Figure 4e. FIGURE S5: Full MSC marker Western blot membranes after imaging (black and white) and ponceau‐stained after protein transfer step to confirm protein loading for Figure 4f. FIGURE S6: Substrate stiffness does not have a significant effect on the anti‐inflammatory effects of iMSC EVs. Levels of pro‐inflammatory cytokines (a) IL‐6 and (b) TNF‐α in conditioned media of RAW264.7 cells treated with 5E9 EVs/mL in an LPS‐stimulated mouse macrophage inflammatory assay, quantified by an ELISA. Dexamethasone (dex) served as a positive control to reduce inflammation. All values expressed as mean ± SD. Data are representative of at least three independent experiments (n = 3). Statistical significance was de [file BTM2-10-e10743-s001.docx]

**Mesenchymal stem cell extracellular vesicle vascularization bioactivity and production yield are responsive to cell culture substrate stiffness**

Emily H. Powsner, Stephanie M. Kronstadt, Kristin Nikolov, Amaya Aranda, Steven M. Jay

**SUPPORTING INFORMATION**


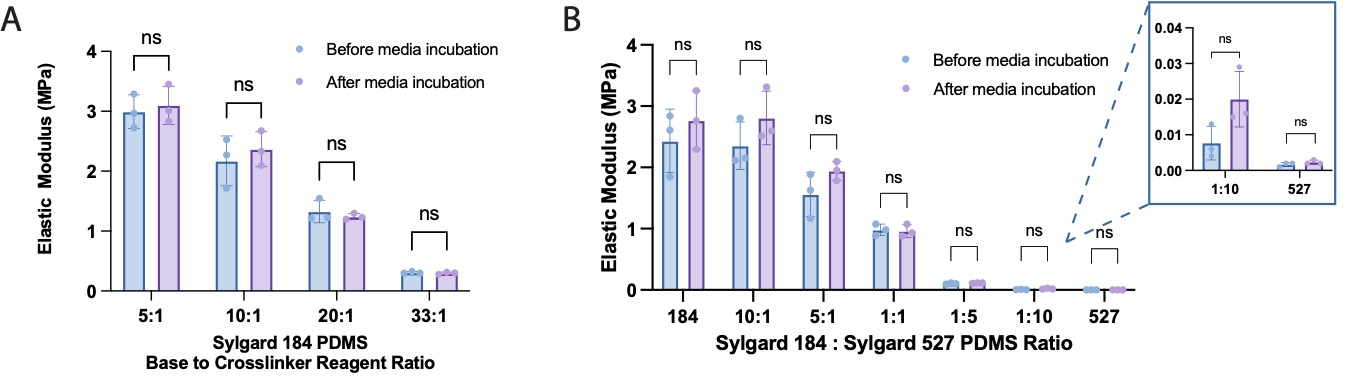


**Figure S1: Incubation in media does not significantly affect the elastic modulus of the PDMS substrates.** Elastic modulus of the A) Sylgard 184 PDMS devices and B) mixed Sylgard 184 and Sylgard 527 PDMS devices before and after being incubated in DMEM a 37ºC for 4 days. All values expressed as mean ± SD. All data are representative of at least 2 independent experiments (n=2). Statistical significance was determined by t-test; *ns = not significant.*

**
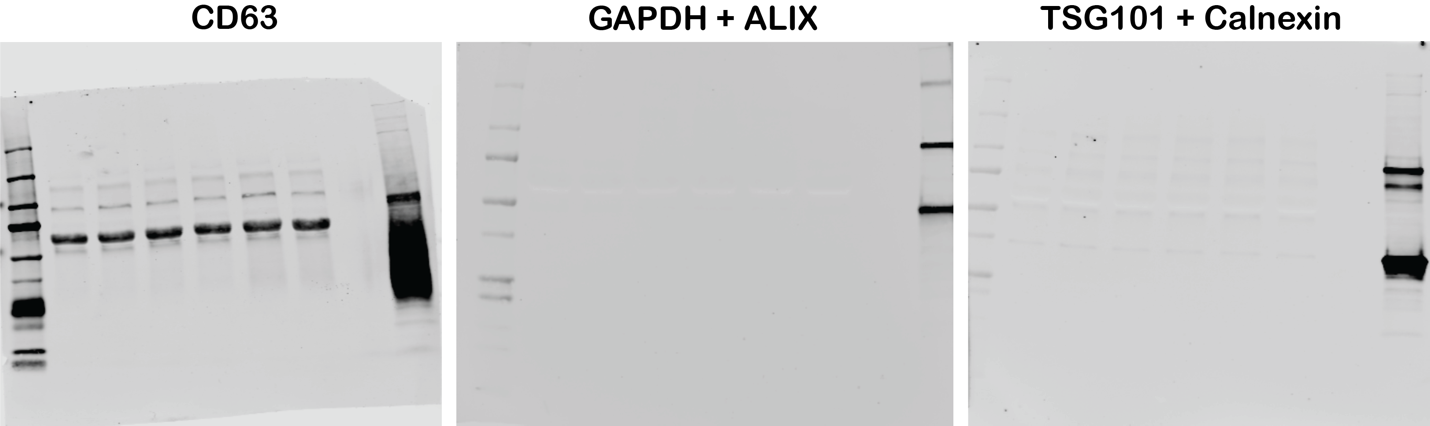
**

**Figure S2: Full EV marker Western blot membranes after imaging (black and white) for Fig. 1D.**

******

**Figure S3: Mixed Sylgard 184 and Sylgard 527 PDMS substrate characterization.** A) Elastic moduli of stiffness devices made with varying ratios of Sylgard 184 and Sylgard 527 PDMS. Values expressed as mean ± SD (n=4). B) Absorbance values from a CCK8 assay indicating cell viability over 4 days. Values expressed as mean ± SEM (n=2). C) Size distribution from NTA of EVs from BM-MSCs seeded on flasks or each substrate made with different ratios of Sylgard 184 and Sylgard 527. Statistical significance was determined by ANOVA; **p<0.05, ***p<0.001, ****p<0.0001.
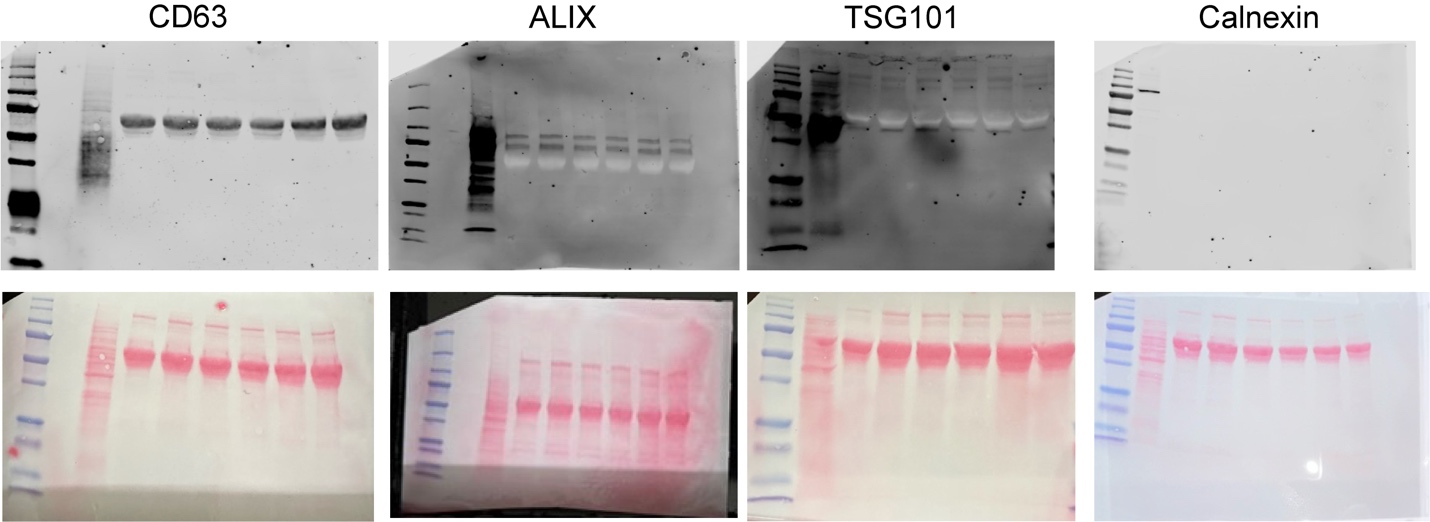
*

**Figure S4: Full EV marker Western blot membranes after imaging (black and white) and ponceau-stained after protein transfer step to confirm protein loading for Fig. 4E.**

**
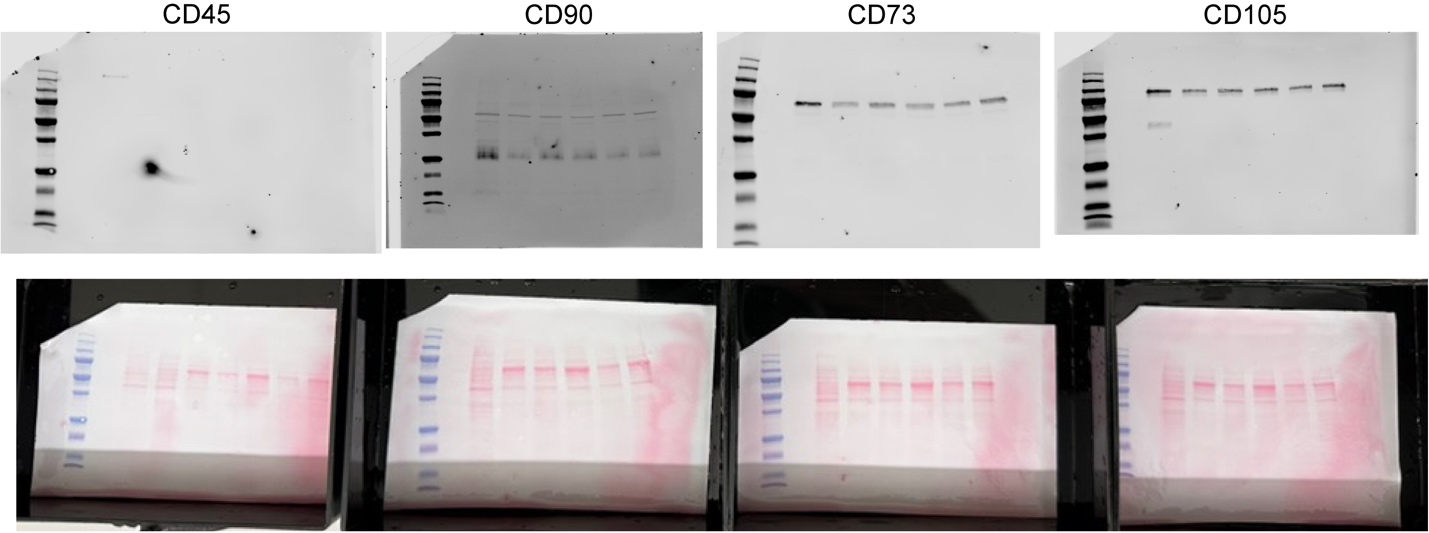
**

**Figure S5: Full MSC marker Western blot membranes after imaging (black and white) and ponceau-stained after protein transfer step to confirm protein loading for Figure 4F.**

**
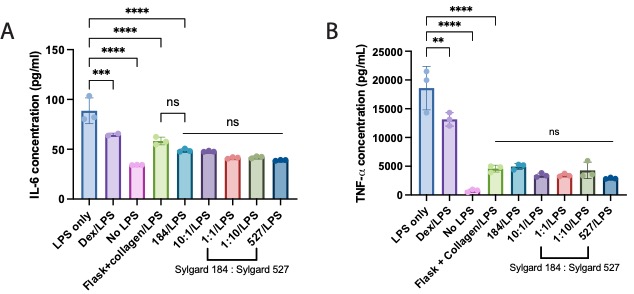
**

**Figure S6: Substrate stiffness does not have a significant effect on the anti-inflammatory effects of iMSC EVs.** Levels of pro-inflammatory cytokines A) IL-6 and B) TNF-alpha in conditioned media of RAW264.7 cells treated with 5E9 EVs/mL in an LPS-stimulated mouse macrophage inflammatory assay, quantified by an ELISA. Dexamethasone (dex) served as a positive control to reduce inflammation. All values expressed as mean ± SD. Data are representative of at least 3 independent experiments (n=3). Statistical significance was determined by ANOVA; *ns = not significant, **p<0.01, ***p<0.001, ****p<0.0001.*

| **Gene** | **Forward** | **Reverse** |
| --- | --- | --- |
| ICAM1 | AGCGGCTGACGTGTGCAGTAAT | TCTGAGACCTCTGGCTTCGTCA |
| CCL2 | AGAATCACCAGCAGCAAGTGTCC | TCCTGAACCCACTTCTGCTTGG |
| HMOX1 | CCAGGCAGAGAATGCTGAGTTC | AAGACTGGGCTCTCCTTGTTGC |
| PTGS2 | CGGTGAAACTCTGGCTAGACAG | GCAAACCGTAGATGCTCAGGGA |

**Table S1:** qPCR primers used for validation of angiogenesis-related genes from the mRNA array.
